# Pooled analysis of efficacy and safety of ureteral stent removal using an extraction string

**DOI:** 10.1097/MD.0000000000017169

**Published:** 2019-09-13

**Authors:** Xujie Sun, Liying Dong, Tao Chen, Zhongyi Huang, Xuebao Zhang, Jitao Wu, Chunhua Lin, Yuanshan Cui

**Affiliations:** a Department of Urology, Qingdao Chengyang People's Hospital, Qingdao; b Department of Urology, Yantai Yuhuangding Hospital Affiliated to Qingdao University, Yantai; c Jimo District Wenquan Hospital, Qingdao; d Department of bid and tender office, Weihai municipal Hospital, Weihai, Shandong; e Department of Urology, Beijing Tian Tan Hospital, Capital Medical University, Beijing, China.

**Keywords:** pooled analysis, randomized controlled trial, removal, string, ureteral stent

## Abstract

**Objective:**

We conducted a Pooled analysis to investigate the efficacy and safety of ureteral stent removal using an extraction string.

**Methods:**

A systematic review was performed by using the Preferred Reporting Items for Systematic Reviews and Pooled analyses. The sources including EMBASE, MEDLINE, and the Cochrane Controlled Trials Register were retrieved to gather randomized controlled trials of ureteral stent removal using an extraction string. The reference of included literature was also searched.

**Results:**

Four randomized controlled trials containing a amount of 471 patients were involved in the analysis. We found that the ureteral stent removal using an extraction string group had a greater decrease of visual analog scale (VAS) (Mean difference (MD) −1.40, 95% confidence interval (CI) −1.99 to −0.81, *P* < .00001) compared with the no string group. The string group did not show a significant differences in Ureteric Stent Symptom Questionnaire (USSQ) (*P* = .15), general health (*P* = .77), stent dwell time (*P* = .06), and urinary tract infection (UTI) (*P* = .59) with exception of stent dislodgement (Odds Ratio (OR) 10.36, 95% CI 2.40 to 44.77, *P* = .002) compared with the no string group.

**Conclusions:**

Ureteral stent removal by string significantly provides less pain than those by cystoscope for patients without increasing stent-related urinary symptoms or UTI. However, this must be balanced against a risk of stent dislodgement and, hence, may not be a good option in all patients.

## 1 Introduction

As the development of endoscopic technology, the indications for retrograde endoscopic therapy to manage urolithiasis have expanded. These endourologic advancements have brought about not only less invasiveness but also higher stone-free rates for patients with urolithiasis.^[1]^ Auge and colleagues reported that 80% of urologists placed a stent after uncomplicated ureteroscopy for stone disease.^[2]^ And most urologists actually insert the stent to avoid stressful emergencies and allow it to remain for 1 to 2 weeks after ureteroscopy.^[2]^ However, importance should also be attached to the quality of life (QoL) of patients as urolithiasis is a benign disease. Previous studies have shown that placing a ureteric stent increases postoperative patient morbidity and negatively affects the patient's QoL.^[3]^ Besides, the additional suffering due to cystoscopic extraction is even more painful. Previous studies have shown that cystoscopy remains a potentially painful procedure, after which gross hematuria, urinary frequency, and dysuria can occur more frequently than expected.^[4]^

Current ureteral stents are manufactured with a string attached to the distal end, allowing for removal without cystoscopy, which may lead to a improvement of patient's QoL. The method is advantageous, but there is wide variability in its clinical application. The rationale behind this is thought to be due to concerns over perceived risks, including increased lower urinary tract symptoms (LUTS) from string irritation, stent dislodgement, infection, stent retention due to patients forgetting to remove stents, broken strings, and lack of strong evidence relating to its safety and tolerability.^[5,6,7]^ A systematic review published in 2016 only included a small number of sample size and lacked of well-designed multicenter randomized controlled trials (RCTs), which resulted in insufficient evidence for the conclusion.^[8]^

Due to paucity in the available literature, we conducted a Pooled analysis of RCTs to evaluate the efficacy and safety of ureteral stent removal using an extraction string.

## 2 Materials and methods

### 2.1 Study protocol

This study was implemented by following the Preferred Reporting Items for Systematic Reviews and Meta-Analyses (PRISMA) checklist.^[9]^ Only randomized controlled studies were included in our study. Observational studies, editorials, commentaries, and review articles were excluded. The abstracts of conference were also excluded. If there was more than 1 publication resulting from the same patient cohort, the most recent publication would be used to analyze.

### 2.2 Information sources and literature search

Based on databases including MEDLINE (1996 to April 2019), EMBASE (1999 to April 2019) and the Cochrane Controlled Trials Register, we did a comprehensive retrieval to analyze the efficacy and safety of ureteral stent removal using an extraction string. The subject headings and text-word terms were as follows: “ ureteral stent, removal, string, and randomized controlled studies”. The study only included published literature with restriction on English language articles. If necessary, authors of the article retrieved were communicated to provide more accurate data for their research. Meanwhile, our study searched for published systematic reviews and other key references. Two investigators screened independently titles and abstracts to identify studies that met the inclusion criteria. When the abstract was insufficient to determine if the study met inclusion criteria, full-text review would be required.

### 2.3 Inclusion criteria and trial selection

Inclusion criteria was as follows:

1. ureteral stent removal using an extraction string was involved;
2. full-text could be acquired;
3. the data provided by the article was valid and valuable, mainly involving the number of cases and valuable results of each indicator;
4. The method of article was a randomized controlled trial;
5. Each study might be added to our study if a group of participants took part in in multiple studies.

**Figure 1:**
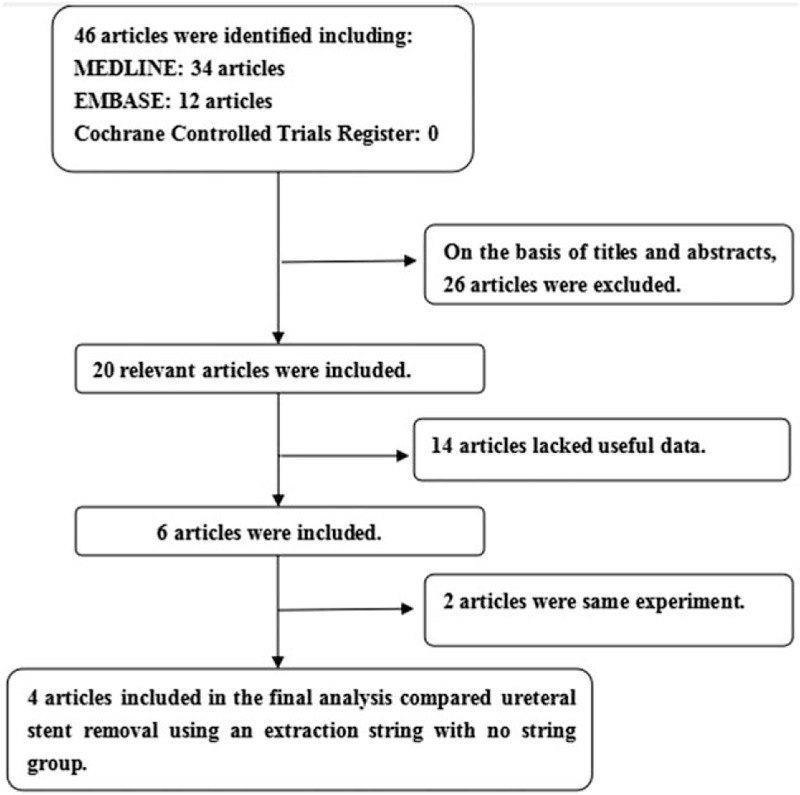
A flow diagram of the study selection process.

The PRISMA diagram of selection was shown in Figure 1.

### 2.4 Quality assessment methods

Our study classified the quality of each study by Jadad scale.^[10]^ Additionally, some measurable methods of assessment were used to assess the quality of the individual studies, including distributive method, concealing distributive process, blindness of process, results of loss to follow and whether there is calculation of sample size or intention-to-treat (ITT) analysis. Studies were graded in line with the principles which derived from the *Cochrane Handbook for Systematic Reviews of Interventions v5.10.*^[11]^ Each RCT was allotted according to following quality classification standards:

- Satisfying almost all of the quality criteria, study would be considered to have a low probability of bias;
- Satisfying the partial quality criteria or unclear, the study was thought of having a secondary probability of bias; or
- Satisfying bare quality criteria, the study was considered to have a high probability of bias.

All authors participated in the assessment of each RCT, eventually everyone agree with the results. All reviewer independently assessed whether the study fitted into the criteria. Any discrepancies were recorded, discussed, and settled among authors.

### 2.5 Data extraction

Based on predetermined criteria, 2 authors independently extracted relevant data from each article. The measurable data was extracted from included studies:

- Abbreviations of first author’ name;
- Published time;
- Country of study;
- Technique received;
- The type of method;
- Number of participants;
- Mean age;
- Data on visual analog scale (VAS), Ureteric Stent Symptom Questionnaire (USSQ), general health, stent dwell time, urinary tract infection and stent dislodgement.

No ethical approval was required for this study.

### 2.6 Statistical analyses and meta-analysis

RevMan version 5.3.0 (Cochrane Collaboration, Oxford, UK)^[11]^ was used to the analysis of data. Fixed or random effects models were applied to assess the study. Mean difference (MD) were applied to evaluate continuous data and odds ratio (OR) for dichotomous results with the corresponding 95% confidence interval [CI].^[12]^ If *P* value > .05, the study was homogeneous, and fixed-effect model was used in our study. The study analyzed variance by Tau^2^ and inconsistency by using *I*^2^ statistic that reflected the proportion of heterogeneity in data analysis. A random effect model would be used for results when the *I*^2^ value is greater than 50% and has significant heterogeneity. If *P* value was less than .05, the result was considered to have statistically significant.

## 3 Results

### 3.1 Study selection process, search results, and characteristics of the trials

Our search found 46 articles by retrieving 3 databases. Screening abstracts and titles, we excluded 26 articles. For remaining 20 articles, 14 articles were excluded because of lack of available data and 2 articles were excluded due to the same experiment (details in Fig. 1). Finally, 4 articles containing 4 RCTs^[5,13–15]^ were involved to evaluate the efficacy and safety of ureteral stent removal using an extraction string. The details of 4 articles were listed in Table 1. Patients with ureteral stent removal using an extraction string included in each study showed similar evaluation index.

**Table 1:**
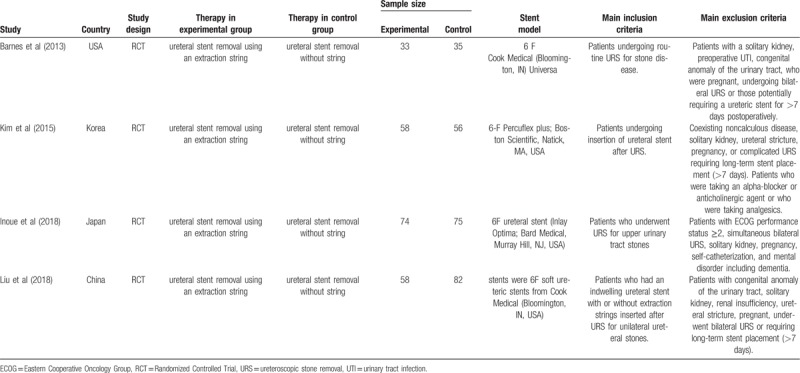
The details of individual study.

### 3.2 Risk of bias in studies

**Figure 2:**
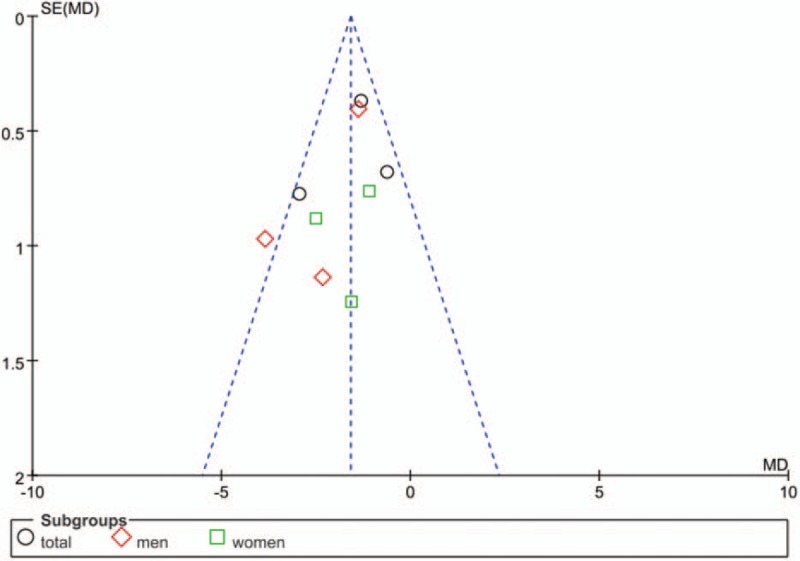
Funnel plot of the studies represented in our analysis. MD = mean difference, SE = standard error.

All studies included in the analysis were random control study. All studies had a appropriate calculation of sample size and no study showed an intention-to-treat analysis. All of the included studies demonstrated a higher quality with Jadad scores rating A (Table 2). The plot was highly symmetrical and 4 squares were contained in the large triangle, and no obvious evidence of bias was found (Fig. 2).

**Table 2:**
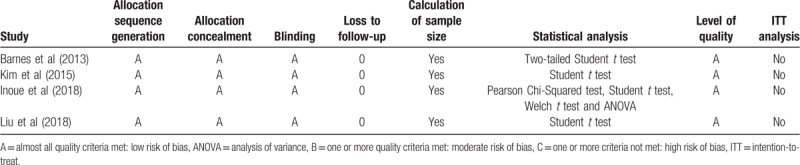
Quality assessment of individual study.

### 3.3 Primary outcomes

#### 3.3.1 VAS

**Figure 3:**
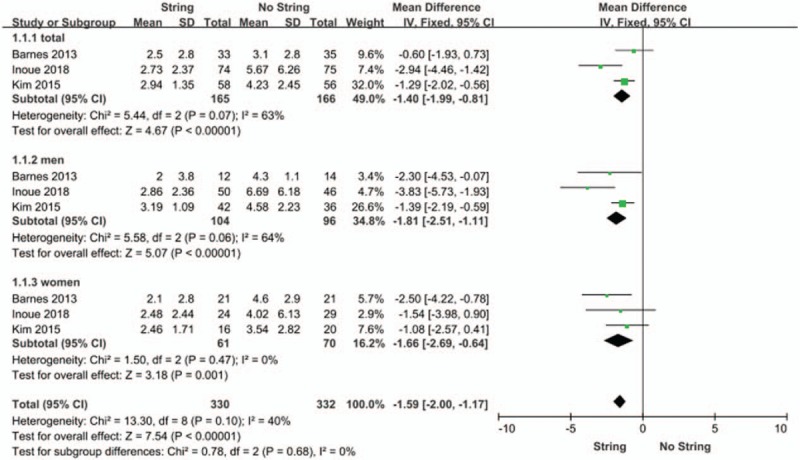
Forest plots showing changes in VAS. CI = confidence interval, IV = inverse variance, SD = standard deviation, VAS = visual analog scale.

Three RCTs gathering a total of 331 patients (165 in the string group and 166 in the no string group) contributed to access VAS data. The forest plot demonstrated that the string group had a lower VAS score (MD −0.14, 95% CI −1.99 to −0.81, *P* < .00001) (Fig. 3) compared with the no string group. Besides, the VAS scores for males and females were both significantly less in the string group (*P* < .00001 and *P* < .001) (Fig. 3).

#### 3.3.2 USSQ

**Figure 4:**
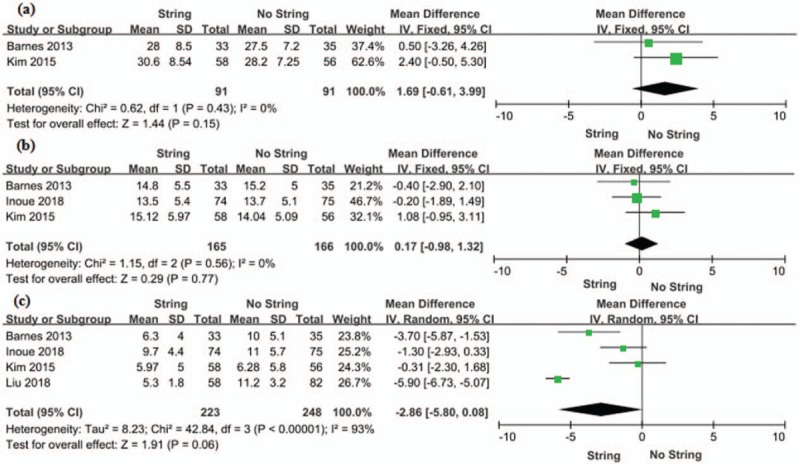
Forest plots showing changes in (a) USSQ, (b) General health and (c) Stent dwell time. CI = confidence interval, IV = inverse variance, SD = standard deviation, USSQ = Ureteric Stent Symptom Questionnaire.

Two RCTs containing a total of 182 patients (91 in the string group and 91 in the no string group) included data on the USSQ. The forest plots showed a MD of 1.69 and 95% CI of −0.61 to 3.99 (*P* = .15) (Fig. 4). We found no statistically significant between string group and no string group in the USSQ.

#### 3.3.3 General health

Three RCTs evaluated the general health with a sample of 331 patients (165 in the string group and 166 in the no string group). The forest plots showed a MD of 0.17 and 95% CI of −0.98 to 1.32 (*P* = .77) (Fig. 4). No statistically significant was found between string group and no string group in general health.

#### 3.3.4 Stent dwell time

Four RCTs included data on the stent dwell time, gathering 471 patients (223 in the string group and 248 in the no string group). The model did not show a marked differences between the 2 groups in the duration of stent dwell time (MD −2.86, 95% CI −5.80 to 0.08, *P* = .06) (Fig. 4).

#### 3.3.5 UTI

**Figure 5:**
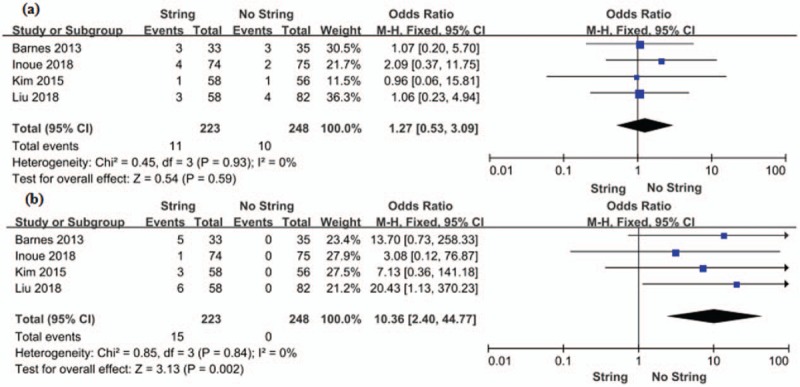
Forest plots showing changes in (a) UTI and (b) Stent dislodgement. CI = confidence interval, MH = mantel haenszel, UTI = urinary tract infection.

Four RCTs with a sample of 471 patients (223 in the string group and 248 in the no string group) evaluated the rates of UTI. The study showed that there is no statistically significant difference between string group and no string group in the incidence of UTI (OR 1.27, 95% CI 0.53 to 3.09, *P* = .59) (Fig. 5).

#### 3.3.6 Stent dislodgement

Four RCTs evaluated the stent dislodgement with a sample of 471 patients (223 in the string group and 248 in the no string group). The fixed-effects estimate of OR was 10.36, and the 95% CI was 2.40 to 44.77 (*P* = .002) (Fig. 5). This result indicated that the risk of stent dislodgement was higher in the string group compared with no string group.

## 4 Discussion

Ureteral stent has been used to facilitate urinary drainage to bladder since 1960s.^[16,17]^ Although benefits in certain patients are clear, indwelling stent present their own set of problems to the patients while in situ and subsequently during their removal. The conventional ureteral stent removal usually requires an elective appointment slot, nursing, medical staff provision, and sometimes potentially even a general anaesthesia. Equipment is also needed, such as a cystoscope, fuid irrigation, camera stack, and stent graspers. Cystoscopy itself is associated with a small risk of morbidity.^[19]^ Besides, travelling to and from the hospital for multiple appointments can be cumbersome and costly for the bulk of patients.

We made this meta-analysis from 4 high quality RCTs including 471 participants to compare the ureteral stent removal using an extraction string with conventional method. Visual Analogue Scale/Score (VAS), this method is more sensitive and comparable. Draw a 10 cm horizontal line on the paper. One end of the horizontal line is 0, indicating no pain; the other end is 10, indicating severe pain; the middle part indicates different degrees of pain. The ureteral stent symptom questionnaire (USSQ), a psychometrically valid measure to evaluate symptoms and impact on quality of life of ureteral stents. Compared with conventional cystoscope method, using an extraction string to removal the ureteral stent had a greater decrease of VAS. Besides, patients with the use of extraction string did not show a significant difference in USSQ, general health, stent dwell time and UTI.

Besides, Kim et al^[14]^ did a randomized controlled study focused on evaluating patients’ preference for ureteral stent removal using an extraction string. As results, they found that most patients preferred removal of the ureteral stent using an extraction string.

Previous systematic review published by Oliver et al^[8]^ found that overall stent dwell time was lower in patients who had their stents removed via extraction strings, which is different from our conclusion. Because of the inclusion of case-control study (CCS) and cohort study, strength of evidence of previous article is relatively weak. Inoue et al^[15]^ and his colleagues reported that ureteral stent removal by string after ureteroscopy significantly provides less pain than those by cystoscope for male patients but not for females. This is also different from our subgroup analysis. Besides, no meta-analysis has been published with respect to this question so far. On all accounts, more high-quality RCTs with suitable study cohorts are needed to confirm our findings.

In respects of stent dislodgement, we found that the string group had a higher rate of the disadvantage compared with no string group. Althaus et al^[18]^ reported that when stratified by gender, 5.3% of men and 24.4% of women with a stent string experienced stent dislodgment (*P* = .013) and women experienced stent dislodgment 4-fold more often than men. The higher rate of stent dislodgment in women may be related to the shorter urethral length or incidentally tugging at the stent string when bathing or voiding. So, we recommend that patients pay great attention and do not tug the stent string when taking a bath or voiding.

Reducing healthcare costs is another advantage of stent extraction string. Barnes et al^[5]^ conducted the trial estimated avoiding the need for second hospital visit and cystoscopy for stent removal resulted in savings in their hospital. Bockholt et al^[19]^ report an estimated $1300/ patient cost associated with cystoscopic stent removal, which would be avoided by patients performing home stent extraction using strings. Liu et al^[14]^ also demonstrated that patients with extraction string had less costly (8.97 ± 3.07 vs 455 ± 0 CNY, *P* = .001) for ureteral stent removal. And the overall cost of patients without an extraction string was significantly more than in patients with an extraction string (86.7 ± 167.7 vs 507.9 ± 147.8 CNY, *P* = .008).

This pooled-analysis includes studies which are all findings from randomized double-blind, placebo-controlled trials. According to the quality-assessment scale that we developed, the quality of the individual studies in the pooled analysis was conforming. The results of this analysis acquire great importance from scientific standpoint, but also in the everyday clinical practice. However, the number of included studies were not many. Selection bias, subjective factors, and publication bias may also affect the final results of our study. One limitation of our findings is some variables, such as stone size, stone location, the skill and experience of the operating surgeon and efficacy of perioperative care. In addition, unpublished studies’ data were not included in the analysis. These factors may have resulted in a bias. More high-quality trials with larger samples are proposed to learn more about the efficacy and safety of ureteral stent removal using an extraction string.

## 5 Conclusion

Ureteral stent removal by string significantly provides less pain than those by cystoscope for patients without increasing stent-related urinary symptoms or UTI. However, this must be balanced against a risk of stent dislodgement and, hence, may not be a good option in all patients.

## Author contributions

**Data curation:** Xujie Sun, Liying Dong.

**Formal analysis:** Xujie Sun, Liying Dong.

**Resources:** Zhongyi Huang, Xuebao Zhang.

**Software:** Zhongyi Huang, Xuebao Zhang, Jitao Wu.

**Supervision:** Jitao Wu, Chunhua Lin, Yuanshan Cui.

**Visualization:** Jitao Wu, Yuanshan Cui.

**Writing – original draft:** Jitao Wu.

**Writing – review & editing:** Tao Chen, Chunhua Lin, Yuanshan Cui.
